# Use of a Diagnostic Score to Prioritize Computed Tomographic (CT) Imaging for Patients Suspected of Ischemic Stroke Who May Benefit from Thrombolytic Therapy

**DOI:** 10.1371/journal.pone.0165330

**Published:** 2016-10-21

**Authors:** Wen Yea Hwong, Michiel L. Bots, Sharmini Selvarajah, L. Jaap Kappelle, Zariah Abdul Aziz, Norsima Nazifah Sidek, Ilonca Vaartjes

**Affiliations:** 1 National Clinical Research Centre, Kuala Lumpur General Hospital, Kuala Lumpur, Malaysia; 2 Julius Center for Health Sciences and Primary Care, University Medical Center Utrecht, Utrecht, the Netherlands; 3 Department of Neurology and Neurosurgery, Brain Center Rudolf Magnus, University Medical Center Utrecht, Utrecht, the Netherlands; 4 Department of Neurology, Hospital Sultanah Nur Zahirah, Terengganu, Malaysia; 5 Department of Pharmacy, Hospital Sultanah Nur Zahirah, Terengganu, Malaysia; "INSERM", FRANCE

## Abstract

**Background:**

A shortage of computed tomographic (CT) machines in low and middle income countries often results in delayed CT imaging for patients suspected of a stroke. Yet, time constraint is one of the most important aspects for patients with an ischemic stroke to benefit from thrombolytic therapy. We set out to assess whether application of the Siriraj Stroke Score is able to assist physicians in prioritizing patients with a high probability of having an ischemic stroke for urgent CT imaging.

**Methods:**

From the Malaysian National Neurology Registry, we selected patients aged 18 years and over with clinical features suggesting of a stroke, who arrived in the hospital 4.5 hours or less from ictus. The prioritization of receiving CT imaging was left to the discretion of the treating physician. We applied the Siriraj Stroke Score to all patients, refitted the score and defined a cut-off value to best distinguish an ischemic stroke from a hemorrhagic stroke.

**Results:**

Of the 2176 patients included, 73% had an ischemic stroke. Only 33% of the ischemic stroke patients had CT imaging within 4.5 hours. The median door-to-scan time for these patients was 4 hours (IQR: 1;16). With the recalibrated score, it would have been possible to prioritize 95% (95% CI: 94%–96%) of patients with an ischemic stroke for urgent CT imaging.

**Conclusions:**

In settings where CT imaging capacity is limited, we propose the use of the Siriraj Stroke Score to prioritize patients with a probable ischemic stroke for urgent CT imaging.

## Introduction

Use of CT imaging is considered the gold standard diagnostic test to distinguish between an ischemic and a hemorrhagic stroke.[1] In areas with inadequate resources, a majority of stroke patients do not have immediate access to CT imaging. A Malaysian national report showed that in 2010, there were 52 machines in 134 public hospitals (39%). In private hospitals, only 44% had direct access to CT imaging.[2] Access to CT imaging is also hindered by the limited availability of ambulances to transport patients. There were 793 functioning ambulances throughout the country in 2010. This equated to a low 0.28 per 10,000 population[3] when compared to the expected 1 for every 10,000 population in high income countries.[4]

This limitation of resources is a major challenge to stroke care in case of time constraints. A delay in diagnosis reduces the eligibility of patients with an ischemic stroke for thrombolytic therapy. Previously, scores to differentiate between the types of stroke were developed based on clinical parameters to accommodate for the shortage of CT machines. Among the scores available include the Siriraj Stroke Score (SSS)[5], the Guys’ Hospital Score or Allen Score[6], the Besson Score[7] and the Greek Stroke Score[8]. Although several validation studies concluded that the scores were not sensitive enough in their detection of hemorrhage to replace CT imaging[9–11], there was a consistent trend of higher predictive abilities of the SSS to rule out a hemorrhagic stroke and thus, increases the chance of detecting an ischemic stroke.[10] Nevertheless, there remains a need to assess the ability of the SSS to identify ischemic stroke patients who are potentially eligible for thrombolytic therapy in the Malaysian population. Instead of replacing CT machines, the SSS is anticipated to act as a triage for prioritization of CT imaging.

This study was therefore conducted to examine the predictive value of the Siriraj Stroke Score to identify patients who are likely to have an ischemic stroke and are potentially eligible for thrombolytic therapy. The identification of these patients would allow prioritization of urgent CT imaging and increase the number of patients that can be treated with thrombolytic therapy.

## Methods

### Cohort Enrollment

Between July 2009 and December 2014, 7592 patients from 14 public hospitals were registered in the Malaysian National Neurology Registry.[12] This is by far the largest and best available representation of the Malaysian population for stroke patients. Public hospitals cover a majority of total hospital admissions in the country; 66.2% of all hospital admissions in 2014 were in public facilities.[13]

Collection of data for this registry follows local routine clinical practice. For the present study, we included patients above 18 years old with signs and symptoms of stroke who arrived in the hospital 4.5 hours or less from ictus. This time frame is in accordance to local and international stroke management guidelines for the eligibility of intravenous thrombolytic therapy.[14,15] We excluded patients who received CT imaging after 15 days from ictus.

Ethical approval was obtained from the Malaysian Ministry of Health’s research ethics committee (ID: NMRR 08-1631-3189). The approval includes data collection and use of data for secondary analysis. With a waiver of informed consent, a public notice is displayed at all participating sites and participants have the option to opt out.

### Types of Stroke

A diagnosis of either a hemorrhagic or a non-hemorrhagic stroke is based on CT imaging. Patients with an intracerebral hemorrhage, subarachnoid hemorrhage (SAH) or ischemia with hemorrhagic transformations were included in the category of hemorrhagic strokes. Ischemic strokes, transient ischemic attacks (TIA) and cerebral venous thrombosis were classified as non-hemorrhagic strokes. More than 90% of patients in the category of non-hemorrhagic strokes consisted of patients with an ischemic stroke, and therefore, the term ‘ischemic stroke’ will be used in subsequent sections for easier interpretation.

CT imaging was interpreted by physicians and subsequently verified by radiologists from the participating hospitals. Stroke scores were not calculated prior to the interpretation of CT imaging. There was no possibility that knowledge of the scores could have an influence on the outcome.

### The Siriraj Stroke Score (SSS)

For each patient, the SSS was calculated. Clinical variables which are needed for this score are listed in Table 1. Some symptoms are not available in the registry. We substituted them with comparable variables: ‘angina’ was replaced with ‘ischemic heart disease’, and instead of ‘intermittent claudication’, we took ‘peripheral artery disease’. Data were obtained via routine examination of patients during their arrival in the hospitals. History of comorbidities was verified with their past medical records.

**Table 1:** Variables in the Original Siriraj Stroke Score.

Variables	Clinical Features	Score	
Level of consciousness	Alert	0	(x2.5)
	Drowsy/Stupor	1	
	Coma/Semi-comatose	2	
Vomiting	No	0	(x2)
	Yes	1	
Headache	No	0	(x2)
	Yes	1	
Diastolic blood pressure (mmHg)			(x0.1)
Atherosclerotic markers (diabetes mellitus, angina or intermittent claudication)	None	0	(x3)
	One or more	1	
Constant			(-12)

Clinicians who developed the original SSS score proposed two cut-off values: <-1 for ischemic stroke and >1 for hemorrhagic stroke.[5] For patients who score between -1 and 1, a distinction between ischemic and hemorrhagic stroke cannot be made. The equation for the original score is:

Logit Probability (Hemorrhagic stroke/Non−hemorrhagic stroke) = 2.5*level of consciousness  + 2*headache  + 2*vomiting  + 0.1*diastolic blood pressure  – 3*atherosclerotic markers – 12

### Statistical Analysis

First, the proportion of missing data was evaluated for each predictor (S1 Table). We carried out multiple imputation with m = 10 (number of imputations) to reduce the extent of bias resulting from missing data.[16] For counts and descriptive statistics, we took the modes of each imputed variable per individual patient. In the event of multiple modes, the mode with highest value was taken. Multiple imputation was conducted with R version 3.1.1.[17]

Second, we fitted the SSS on our dataset. Performance of the predictive ability of the score is evaluated via discrimination: area under the curve (AUC) of receiver operating characteristics (ROC) curves is calculated[18]; and via calibration: the slope and intercept of calibration plots are estimated to assess differences between predicted and observed probabilities[19].

Thirdly, the analysis was extended with updating methods to obtain a ‘best, fitted score’.[20] We recalibrated the intercept and the slope and adjusted the regression coefficients for predictors ‘level of consciousness’ and ‘headache’. Details of the updating methods and operationalization of each predictor are shown in S1 and S2 Tables. Furthermore, performance of the recalibrated score is tested for its internal validity via bootstrapping. With bootstrap analysis, the shrinkage factor and the optimism corrected AUC are calculated.[20]

Lastly, we took different cut-off values of the recalibrated SSS to assess its diagnostic performance in distinguishing between a hemorrhagic and an ischemic stroke. We also attempted to identify possible effect modifications for the score across age categories, sex and cardiovascular risk factors including hypertension, hyperlipidemia and atrial fibrillation. As an additional analysis, we validated the recalibrated score in a cohort where the inclusion time frame was expanded to 6 hours from ictus. This is to accommodate ischemic stroke patients who were potentially eligible for intra-arterial thrombolytic therapy.[14,15]

Statistical analysis was performed using Stata version 13.0.[21]

## Results

### Baseline Characteristics

Of 2176 patients who were included, 57% were males. The mean age was 62 (SD: 12) years (Table 2). Cardiovascular risk factors were common: nearly three quarters of patients had hypertension (73%), 40% with diabetes mellitus and 28% had dyslipidemia. Only 4% of the included patients had a history of atrial fibrillation whereas almost 50% of them were current smokers. Mean blood pressure upon arrival was 171mmHg (SD:36) for systolic and 94mmHg (SD:21) for diastolic. The median door-to-scan time was 4 hours (IQR: 1;14) and the median time taken for patients to arrive in the emergency departments was 2 hours (IQR: 1;3).

**Table 2:** Baseline Characteristics.

Characteristics	Patients in the study
	n = 2176
Mean age(years) ± SD*	62 ± 12
Sex, n (%)	
Male	1247 (57)
Female	929 (43)
Ethnic group, n (%)	
Malay	1816 (83)
Non-Malay	360 (17)
Co-morbidities, n (%)	
Hypertension	1584 (73)
Diabetes Mellitus	861 (40)
Dyslipidemia	615 (28)
Ischemic Heart Disease	310 (14)
Atrial Fibrillation	84 (4)
Previous TIA/stroke events	453 (21)
Life-style factors, n (%)	
Obesity	173 (8)
Smoking status	
Current	1063 (49)
Previous smoker (quit >30 days)	400 (18)
Never	713 (33)
Clinical presentation during admission	
Mean Systolic BP(mmHg) ± SD	171 ± 36
Mean Diastolic BP(mmHg) ± SD	94 ± 21
Mean Pulse rate (bpm) ± SD	84 ± 19
Median oxygen saturation rate (Sp02)% (IQR)^†^	99 (98;100)
Headache	597 (27)
Vomiting	447 (21)
Seizure at onset of stroke	209 (10)
Median NIHSS^‡^ score (IQR)	10 (3;22)
Level of consciousness	
Alert	1402 (64)
Drowsy/Stupor	384 (18)
Semicomatose/Coma	390 (18)
Duration	
Median Time of onset to door (hours) (IQR)	2 (1;3)
Median Time of door to scan (hours) (IQR)	4 (1;14)
Median Time of onset to scan (hours) (IQR)	6 (4;16)

*SD:standard deviation,

^†^IQR: interquartile range,

^‡^NIHSS: National Institute of Health Stroke Scale

### Fitting the Siriraj Stroke Score

Initial calibration of the original SSS showed a poor fit. There was overestimation at higher observed probabilities and underestimation at lower observed probabilities. The recalibrated score, in which the intercept and weights of the individual determinants were adjusted showed a better fit with an AUC of 0.80 (95%CI: 0.78–0.83). Post bootstrap analysis, a shrinkage factor of 0.99 and an optimism for the AUC of 2.18x10^-5^ were found. The optimism-corrected AUC was similar to that of the recalibrated score (results in S1 Fig and S3 Table). After simplification of the coefficients, the equation for the recalibrated score is:

Logit Probability (Hemorrhagic stroke/Non−hemorrhagic stroke) = 1.0*level of consciousness + 0.3*headache + 0.7*vomiting + 0.03*diastolic blood pressure – 1.0* atherosclerotic markers– 4.5

Potential effect modifiers were tested and no significant interactions were found.

### Use of the Recalibrated Siriraj Stroke Score in Prioritization of CT Imaging

In the present cohort, 73% (n = 1594) of the patients had an ischemic stroke. Only one third of these ischemic stroke patients (33%) who were potentially eligible for thrombolytic therapy received CT imaging within the therapeutic window period of 4.5 hours.

Table 3 provides results of the application of the recalibrated SSS for identification of an ischemic stroke. Depending on the choice of cut-off values, results differ. At a threshold of 0 (SSS equal or higher than 0 defines a hemorrhagic stroke), 95% (n = 1519) of the ischemic stroke patients would have been correctly diagnosed, thus prioritized for urgent CT imaging.

**Table 3:** Application of the Recalibrated Siriraj Stroke Score at Different Thresholds.

Cut-off values	Sensitivity with 95% CI (%)	Specificity with 95%CI (%)	PPV^†^ with 95% CI (%)	NPV^†^ with 95% CI (%)	Number of missed ischemic cases (%)	Number of overdiagnosed cases, n (%)	Number of urgent CT imaging (%)
> = -1.5	84 (81–87)	63 (60–65)	45 (42–48)	92 (90–93)	591 (37)	91 (8)	1094 (50)
> = -1.0	74 (70–77)	80 (78–82)	57 (54–61)	89 (87–91)	320 (20)	154 (11)	1428 (66)
> = -0.5	62 (58–66)	90 (89–92)	69 (65–73)	87 (85–88)	158 (10)	223 (13)	1659 (76)
> = 0*	49 (44–53)	95 (94–96)	79 (74–83)	84 (82–85)	75 (5)	300 (16)	1819 (83)
> = 0.5	34 (31–38)	98 (97–99)	88 (83–92)	80 (79–82)	28 (2)	382 (20)	1948 (90)
> = 1.0	19 (16–23)	100^‡^ (99–100)	93 (87–97)	77 (75–79)	8 (0.5)	471 (23)	2057 (95)
> = 1.5	8 (6–10)	100^‡^ (99.5–100)	96 (85–99)	75 (73–77)	2 (0.1)	537 (25)	2129 (98)

*cut-off value used in this study,

^†^PPV: positive predictive value,

^‡^NPV: negative predictive value.

By applying the recalibrated SSS, 83% of the cohort would have been sent for urgent CT imaging. Of those, 84% would have had an ischemic stroke and 16% would have been diagnosed with a hemorrhagic stroke. Around 5% of the ischemic stroke patients would not have immediate CT imaging (Table 3).

Results from the additional analysis where the inclusion time frame was expanded from 4.5 hours to 6 hours (n = 2658) showed similar results to the above (S4 Table).

## Discussion

Despite arriving within the therapeutic time for thrombolytic therapy, two-thirds of the ischemic stroke patients did not receive urgent CT imaging by 4.5 hours. This delay in diagnosis inevitably eliminate their potential eligibility for thrombolytic therapy. With the recalibrated SSS, 95% of patients with an ischemic stroke who were potentially eligible for thrombolytic therapy, could be prioritized for urgent CT imaging.

Our results on the discriminative power of the SSS were comparable with two validation studies who had reported an AUC of 0.78 (95% CI: 0.75–0.82)[22] and 0.80(95% CI not given)[23]. Previous studies including a study of smaller sample size from Malaysia reported that the original SSS has failed to achieve a high sensitivity to detect hemorrhagic stroke.[9–11,24] This is particularly so in Western countries.[25,26] Nevertheless, higher specificities for the score were found. This increases the predictive ability of the score to identify an ischemic stroke. For example, Mwita et al[10] in a systematic review reported consistently higher specificities of the SSS with a range from 65%–99% compared to its corresponding sensitivities in 18 validation studies. This is in agreement with our specificity findings. The specificity for the recalibrated score was further improved with a choice of a different cut-off value. Moreover, parallel to our aims, some of the studies[22,27] supported the use of the higher predictive rule-out ability of the SSS to detect ischemic stroke patients in areas with insufficient resources.

Previous validation studies focused on the ability of the SSS to replace CT imaging.[5,9–11,22] Here, we propose to use this score to prioritize patients who are likely to have an ischemic stroke and are potentially eligible for thrombolytic therapy for urgent CT imaging. Up to the present time, only few hospitals in the country are administering thrombolytic therapy. Reasons for this poor uptake include strict exclusion criteria such as prior use of Vitamin K antagonist, uncertainty over its benefit for elderly patients and patients with mild or very severe stroke and most importantly, a narrow therapeutic window time.[28] Although this is a similar picture globally[29], lack of infrastructure is often an additional barrier to the administration of this therapy. The present study focuses on an aspect which is common in developing regions; time delays resulting from a shortage of CT machines. Other criteria which deny patients from their eligibility for thrombolytic therapy were not taken into account in our selection of the best patients for prioritization of CT imaging.

Establishing a clinical diagnosis of an ischemic stroke with the SSS will thus, allow prioritization of the right patients for the right treatment at the right time. Moreover, there are only 5 clinical variables needed with the SSS; all easily retrieved during initial assessment of patients with symptoms of stroke. The simplicity of this score and its practical application has been commended in several studies.[5,10] Nevertheless, we are aware that the recalibrated SSS may not be easily memorized or applied without a calculator. Future implementation of this score with a possible smartphone application will be useful.

Our study had a sufficient sample size to validate the score. We performed multiple imputations to minimize possible biases from missing data. Moreover, instead of developing a new score, we chose to revise the original SSS by applying updating methods. In this respect, the score was adjusted to the new validated population without losing prior information from the development dataset.[20,30] One limitation of the study is the lack of information on stroke-mimicking diagnoses. Nevertheless, a huge majority of patients with stroke symptoms do have a diagnosis of a stroke. Previous studies showed varying rates of stroke mimics from 9–19%[31–33]. Selection bias from our selection of cohort should therefore be minimal.

There are a few important points to be emphasized with regards to the score. First, there is no intention to deprive patients from CT imaging. All suspected-for-stroke patients should receive CT imaging for diagnostic purposes. Our target patients for prioritization are ischemic stroke patients who reach the hospital within 4.5 hours because there is a tight time constraint to maximize the benefits from thrombolytic therapy. In our cohort, despite having a similar median duration of time taken to arrive at the hospital from stroke onset, patients with hemorrhagic stroke were found to have a shorter door-to-scan time in comparison to ischemic stroke patients. The median door-to-scan time was 2 hours (IQR:1;8) for the former and twice as long for the latter. While we acknowledge the urgency of a surgical procedure for patients who are suspected of a subarachnoid hemorrhage, to our best knowledge, there is no specific window period to perform surgical coiling or clipping.[34,35]

Second, the aim of this score is to guide decisions and not to overrule clinical concerns. Other factors which may influence a physician’s judgment in prioritizing patients who are suspected of a stroke for immediate CT imaging include age, other comorbidities and severity of the condition. Third, this score was recalibrated to the Asian or specifically, the Malaysian population. Differences in terms of prevalence of hemorrhagic stroke and other cardiovascular risk factors between populations may limit the generalizability of the score’s utility. The performance of this recalibrated score should be tested prior to its application in clinical practice for other populations. Fourth and most importantly, at the cut-off value chosen, 5% of ischemic stroke cases were underdiagnosed. We took the threshold of 0 to minimize this proportion of patients and at the same time, finding the best balance between false positives and false negatives. This may be less than ideal but in places that lack infrastructures, choices have to be made and we consider this as the best alternative available. Preferences of choosing specific cut-off values may differ according to the availability of resources in respective regions. With the threshold chosen, we would have to prioritize 83% of patients for urgent CT imaging. While prioritization improves efficiency in hospitals with limited CT machines, foreseeable challenges lie in hospitals without CT machines that require inter-hospital transfers for urgent CT imaging. One would be the capacity to transport these patients to hospitals with CT machines available. Should a shortage in this aspect occur, cut-off values would have to be reduced but, at the expense of a higher proportion of missed ischemic stroke cases for urgent CT imaging. Another relevant point to note is the influence of the duration for inter-hospital transfers on the eligibility for thrombolytic therapy. Having said that, about 50% of the suspected patients in our cohort arrived at the hospital within 2 hours from the onset of their symptoms. Priorities therefore, should be given to these patients once they scored below 0 (suspected ischemic stroke) for immediate stabilization and transfer for urgent CT imaging.

## Conclusions

While the best option is still to have immediate access to CT imaging for every stroke patient, a shortage of CT machines would probably remain as a problem in the near future. Prioritization with the Siriraj Stroke Score aims to reduce the issues of insufficient resources. More importantly, its application is targeted to optimize stroke care especially for ischemic stroke patients with time-dependent therapy.

## Supporting Information

S1 Fig Comparison of Calibration Plots Prior to (original score) and after (recalibrated score) Updating Methods. (TIF)

S1 Table Proportion of Missing Data and Operationalization of Predictors. (DOCX)

S2 Table Updating Methods. (DOCX)

S3 Table Regression Coefficients for Each Predictor by Methods. (DOCX)

S4 Table Application of the Recalibrated Siriraj Stroke Score at Different Thresholds (expanded time window of 6 hours). (DOCX)
