# Supplementary figures and images for: Use of a Diagnostic Score to Prioritize Computed Tomographic (CT) Imaging for Patients Suspected of Ischemic Stroke Who May Benefit from Thrombolytic Therapy
Source: PLoS One. 2016 Oct 21;11(10):e0165330. doi: 10.1371/journal.pone.0165330 (PMC5074585; doi:10.1371/journal.pone.0165330)

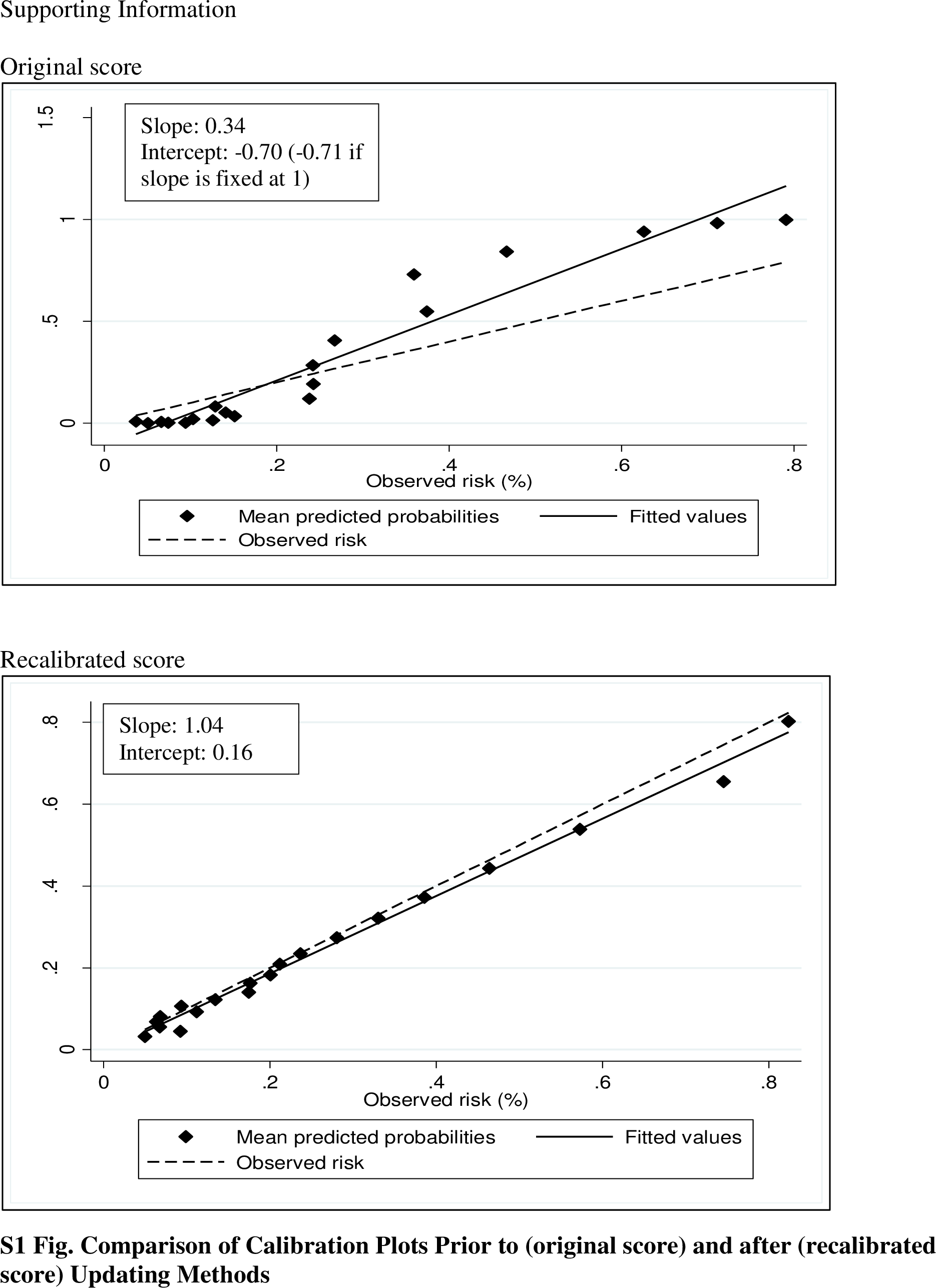

Supplement: S1 Fig — (TIF) [file pone.0165330.s001.tif]
