# Supplementary material for: Use of a Diagnostic Score to Prioritize Computed Tomographic (CT) Imaging for Patients Suspected of Ischemic Stroke Who May Benefit from Thrombolytic Therapy
Source: PLoS One. 2016 Oct 21;11(10):e0165330. doi: 10.1371/journal.pone.0165330 (PMC5074585; doi:10.1371/journal.pone.0165330)
Supplement: S1 Table — (DOCX) [file pone.0165330.s002.docx]

Supporting Information

**S1 Table. Proportion of Missing Data and Operationalization of Predictors**

| Characteristics | Missing data, n (%)* | Type of variable | Operationalization |
| --- | --- | --- | --- |
| Age | 0 (0) | Numerical | measured in years (date of admission is subtracted with date of birth) |
| Sex | 0 (0) | Nominal | dichotomous variable: male and female |
| Co-morbidities |  |  |  |
| Hypertension | 0 (0) | Nominal | documented history of hypertension prior to stroke event: yes and no |
| Diabetes Mellitus | 0 (0) | Nominal | documented history of diabetes mellitus type 2 prior to stroke event: yes and no |
| Dyslipidemia | 0 (0) | Nominal | documented history of hyperlipidemia prior to stroke event: yes and no |
| Ischemic Heart Disease | 0 (0) | Nominal | documented history of ischemic heart disease prior to stroke event: yes and no |
| Atrial Fibrillation | 0 (0) | Nominal | documented history of atrial fibrillation prior to or during stroke event: yes and no |
| Peripheral artery disease | 0 (0) | Nominal | documented history of peripheral artery disease prior to stroke event: yes and no |
| Clinical presentation during admission |  |  |  |
| Diastolic BP | 0 (0) | Numerical | first reading at arrival in the hospital; measured in mmHg |
| Headache | 92 (4) | Nominal | self/bystander reporting of headache upon onset of stroke |
| Vomiting | 70 (3) | Nominal | self/bystander reporting of nausea or vomiting upon onset of stroke |
| Level of consciousness | 694 (32) | Nominal | categorized into 3 levels: 0 for alert and responsive; 1 for arousable by stimulation; and 2 for responding only with motor reflexes/autonomic effects or totally unresponsive |

*denominator for proportions, n=2176
