# Supplementary material for: Use of a Diagnostic Score to Prioritize Computed Tomographic (CT) Imaging for Patients Suspected of Ischemic Stroke Who May Benefit from Thrombolytic Therapy
Source: PLoS One. 2016 Oct 21;11(10):e0165330. doi: 10.1371/journal.pone.0165330 (PMC5074585; doi:10.1371/journal.pone.0165330)
Supplement: S2 Table — (DOCX) [file pone.0165330.s003.docx]

Supporting Information

**S2 Table. Updating methods**

| Methods | Description | Steps |
| --- | --- | --- |
| 0 | Unadjusted score | 1. No adjustment needed |
| 1 | Recalibration of the intercept | 1. A logistic regression was fitted with the linear predictor (LP1)* as the only covariate. The coefficient for the LP1 was fixed at 1 to obtain an intercept. 2. The intercept from the regression was added to the original intercept to obtain a recalibrated intercept. |
| 2 | Recalibration of the intercept and slope | 1. A logistic regression was fitted with the LP1 as the only covariate. 2. All regression coefficients from the LP1 were multiplied with the slope from the logistic regression. 3. The intercept from the LP1 was multiplied with the slope from the logistic regression before it was added to the intercept from the regression. |
| 3 | Adjustment of regression coefficient for one of the predictors | 1. This method was only applicable after method 2 was performed. 2. A logistic regression was fitted with the revised linear predictor from method 2 and each of the predictors separately. The coefficient for the linear predictor was fixed at 1. From each model, predictors were selected with forward selection, starting with the predictor with the largest Wald statistic. 3. Using likelihood ratio test (p<0.05), nested model were tested to assess their added values. Post estimations were not available for multiple imputed dataset and therefore, likelihood ratio test was performed with m=1. 4. Deviation of beta coefficients for predictors “level of consciousness” and “headache” were significant. Bootstrap was performed (n=200 datasets) to obtain a shrinkage factor. 5. Deviation of beta coefficients were multiplied with the shrinkage factor before being added to the recalibrated regression coefficients from Method 2 to form the recalibrated score (LP2)^†^. 6. Intercept was refitted after shrinkage. |
| 4 | Simplifying coefficients | 1. The initial recalibrated score (LP2) was 1.1*level of consciousness + 0.3*Headache + 0.7*Vomiting + 0.03*Diastolic blood pressure – 1.0*Atheroma - 4.4 2. Division and multiplication of the coefficients with any constant resulted in changes to the calibration plots. LP2 was therefore simplified to only 1.0*Consciousness + 0.3*Headache + 0.7*Vomiting + 0.03*Diastolic blood pressure – 1.0*Atheroma - 4.5 |

*LP1 = 2.5*Consciousness + 2*Headache + 2*Vomiting + 0.1*Diastolic blood pressure – 3*Atheroma - 12

^†^LP2= 1.0*Consciousness + 0.3*Headache + 0.7*Vomiting + 0.03*Diastolic blood pressure – 1.0*Atheroma - 4.5
