# Supplementary material for: Use of a Diagnostic Score to Prioritize Computed Tomographic (CT) Imaging for Patients Suspected of Ischemic Stroke Who May Benefit from Thrombolytic Therapy
Source: PLoS One. 2016 Oct 21;11(10):e0165330. doi: 10.1371/journal.pone.0165330 (PMC5074585; doi:10.1371/journal.pone.0165330)
Supplement: S3 Table — (DOCX) [file pone.0165330.s004.docx]

Supporting Information

**S3 Table. Regression Coefficients for Each Predictor by Methods**

|  | Methods | | | |  |
| --- | --- | --- | --- | --- | --- |
| Predictors | 0 | 1 | 2 | 3 | 4 (simplifying coefficients) |
| Consciousness | 2.5 | 2.5 | 0.9 | 1.1 | 1.0 |
| Headache | 2 | 2 | 0.7 | 0.3 | 0.3 |
| Vomiting | 2 | 2 | 0.7 | 0.7 | 0.7 |
| Diastolic blood pressure | 0.1 | 0.1 | 0.03 | 0.03 | 0.03 |
| Atheroma | -3 | -3 | -1 | -1 | -1.0 |
| Constant | -12 | -12.7 | -4.8 | -4.4 | -4.5 |
| Slope | 0.34 | 0.34 | 1.03 | 1.00 | 1.04 |
| Intercept | -0.70 (-0.71 if slope is fixed at 1) | -0.47 | 0.39 | -0.05 | 0.16 |
| Shrinkage factor | - | - | - | 0.99 | 0.99 |
| Area under the curve (AUC) with 95% CI | 0.80 (0.77-0.83) | 0.80 (0.77-0.83) | 0.80 (0.77-0.83) | 0.80 (0.78-0.83) | 0.80 (0.78-0.83) |
