# Supplementary material for: Use of a Diagnostic Score to Prioritize Computed Tomographic (CT) Imaging for Patients Suspected of Ischemic Stroke Who May Benefit from Thrombolytic Therapy
Source: PLoS One. 2016 Oct 21;11(10):e0165330. doi: 10.1371/journal.pone.0165330 (PMC5074585; doi:10.1371/journal.pone.0165330)
Supplement: S4 Table — (DOCX) [file pone.0165330.s005.docx]

Supporting Information

**S4 Table. Application of the Recalibrated Siriraj Stroke Score at Different Thresholds (expanded time window of 6 hours)**

| Cut-off values | Sensitivity with 95% CI (%) | Specificity with 95%CI (%) | PPV^†^ with 95% CI (%) | NPV^†^ with 95% CI (%) | Number of missed ischemic cases (%) | Number of overdiagnosed cases, n (%) | Number of urgent CT imaging (%) |
| --- | --- | --- | --- | --- | --- | --- | --- |
| >=-1.5 | 85 (82-87) | 63 (61-66) | 45 (43-48) | 92 (91-93) | 716 (37) | 107 (8) | 1349 (51) |
| >=-1.0 | 75 (71-78) | 80 (78-82) | 58 (54-61) | 90 (88-91) | 388 (20) | 176 (10) | 1746 (66) |
| >=-0.5 | 62 (59-66) | 90 (88-91) | 69 (65-72) | 87 (85-88) | 200 (10) | 263 (13) | 2021 (76) |
| >=0* | 48 (44-52) | 95 (94-96) | 77 (73-81) | 84 (82-85) | 100 (5) | 365 (16) | 2223 (84) |
| >=0.5 | 34 (31-38) | 98 (97-98) | 84 (79-88) | 81 (79-82) | 46 (2) | 460 (19) | 2372 (89) |
| >=1.0 | 20 (17-23) | 99 (99-100) | 90 (84-94) | 78 (76-79) | 16 (1) | 558 (22) | 2500 (94) |
| >=1.5 | 10 (7-12) | 100 (99-100)^‡^ | 93 (85-98) | 76 (74-77) | 5 (0.3) | 632 (24) | 2585 (97) |

*cut-off value used in this study, ^†^PPV: positive predictive value, ^‡^NPV: negative predictive value, n=2658
